# Interpretable Probabilistic Identification of Depression in Speech

**DOI:** 10.3390/s25041270

**Published:** 2025-02-19

**Authors:** Stavros Ntalampiras

**Affiliations:** Department of Computer Science, University of Milan, 20133 Milan, Italy; stavros.ntalampiras@unimi.it

**Keywords:** medical acoustics, audio pattern recognition, universal background modeling, hidden Markov models, mental health, depression, explainable AI, interpretable AI

## Abstract

Mental health assessment is typically carried out via a series of conversation sessions with medical professionals, where the overall aim is the diagnosis of mental illnesses and well-being evaluation. Despite its arguable socioeconomic significance, national health systems fail to meet the increased demand for such services that has been observed in recent years. To assist and accelerate the diagnosis process, this work proposes an AI-based tool able to provide interpretable predictions by automatically processing the recorded speech signals. An explainability-by-design approach is followed, where audio descriptors related to the problem at hand form the feature vector (Mel-scaled spectrum summarization, Teager operator and periodicity description), while modeling is based on Hidden Markov Models adapted from an ergodic universal one following a suitably designed data selection scheme. After extensive and thorough experiments adopting a standardized protocol on a publicly available dataset, we report significantly higher results with respect to the state of the art. In addition, an ablation study was carried out, providing a comprehensive analysis of the relevance of each system component. Last but not least, the proposed solution not only provides excellent performance, but its operation and predictions are transparent and interpretable, laying out the path to close the usability gap existing between such systems and medical personnel.

## 1. Introduction

Unfortunately, there is a significant part of the population that suffers from mental health disorders, resulting in a substantial decrease in their quality of life [1]. At the same time, such illnesses are associated with a considerable economic impact, which is higher than cancer, cardiovascular, and respiratory diseases [2]. However, governmental budgets are typically higher for such diseases with respect to mental illnesses. At present, the diagnosis of such conditions is first and foremost based on reports produced either by the subjects themselves, i.e., self-assessment, or their caregivers. More often than not, such reports are subjective and biased, increasing the probability of an erroneous condition assessment [3]. Importantly, there is a great need to objectify such a diagnosis process while making it available over time so that subjects can be treated on a regular basis and receive evidence-based therapy, especially where such psychological services are limited or even nonexistent. Such periodic and methodological assessments would result in improved therapeutics, decreasing the associated negative consequences. The diagnostic procedure clearly depends significantly on the availability of skilled professionals and their specialized knowledge in the particular mental health condition. Consequently, it becomes a time-intensive process accompanied by an excessively high cost.

A different diagnostic tool that alleviates these constraints involves the development of audio pattern recognition systems that analyze speech signals and draw inferences about the individual’s health status. Certainly, the objective is not to substitute for the expertise of a medical professional but rather to create a supportive tool that can offer an initial diagnosis and, potentially, expedite the assessment process. Various modalities have been used for identifying depression, such as visual, EEG, etc. [4,5,6,7], while this work focuses exclusively on the analysis of speech signals.

Among the various mental disorders, there are several works concentrating on processing speech for detecting depression [8,9]. There, both handcrafted and features learned automatically in conjunction with generative and discriminative classifiers have been employed [10,11,12,13]. A systematic review is out of the scope of this work, while the interested reader may consult the systematic survey presented in [3]. By collectively analyzing the related literature, several interesting observations can be made regarding existing gaps: 1. there is not a standardized experimental protocol permitting reproducible research and reliable comparison of different approaches; 2. temporal modeling approaches have not been systematically considered; 3. interpretation of the model predictions has not been thoroughly considered by the research community; and 4. the majority of the existing approaches consider small-sized datasets, where overfitting may occur and limit their generalization across corpora of diverse characteristics.

This work argues that, given the increased difficulty of the task—where even medical experts need several sessions/weeks to provide a secure assessment—along with the limited data availability, automated end-to-end feature learning does not comprise a feasible path yet. As such, and considering the need for fully interpretable predictions, handcrafted features are designed to capture specific properties of the signal’s structure. Unfortunately, explainability has not been systematically explored in such a critical scenario, let alone the interaction between the AI system and medical experts. In addition, in detecting speech patterns indicative of healthy/depressed subjects, it is of paramount importance to take into account their evolving structure. This article covers the aforementioned gaps via a pattern modeling scheme explicitly encoding existing temporal dependencies while offering fully interpretable predictions aimed at closing the usability loop present between AI-based systems and medical experts.

We propose to use a multifaceted feature set combined with a universal data modeling approach. More specifically, the feature set is composed of (a) Mel-Frequency Cepstral Coefficients (MFCC), (b) Teager Energy Operator (TEO), and (c) Fundamental frequency and harmonic ratio.

The first one is the log Mel-scaled spectrogram summarized using the discrete cosine transform. The second one consists of areas included under the TEO autocorrelation envelope extracted after a critical band-based analysis, as described in [14]. Last but not least, the third set captures the periodic nature of the available speech signals. As such, these features capture complementary characteristics of the audio structure and their combined use is be beneficial for identification of depression in speech. These feature sets are juxtaposed and fed to a universal probabilistic modeling scheme based on Hidden Markov Models (HMM).

Initially, a universal HMM is constructed by employing a balanced subset of the training data, including recordings coming from both classes. At a second stage, using the remaining data of each class, we create adapted versions of the universal model to represent each class. Thus, the class(es) use as a basis a model whose parameters have been learned using a larger amount of data and, as such, comprise finer estimations with respect to using a more limited set. Importantly, we designed a novel data selection scheme, taking into account the perceptual nature of the present problem for determining the model training and adaptation data. This work employs the publicly available Androids dataset [15], which has been specifically designed for capturing depression in speech and presents several advantages over existing ones, i.e., it was annotated by professional psychiatrists without considering self-assessment questionnaires, it was recorded at the location where patients are actually treated, and it comes with a standardized experimental protocol.

Last but not least, the proposed approach was built considering explainability by design, where its operation is fully transparent and takes into account the requirements of such a medical setting while being able to interact with the medical experts. Such a design paradigm examines and addresses problem-related characteristics without seeking to decipher the operation of black-box models at a post-evaluation stage.

The rest of this work is organized as follows: Section 2 formalizes the problem, while Section 3 describes in detail the proposed pipeline, focusing on the feature extraction and pattern recognition algorithms. Subsequently, Section 4 thoroughly examines the efficacy of the proposed solution and contrasts it with existing solutions. Section 5 underlines the interpretation capabilities of the present solution, while Section 6 reports and comments on the most important findings along with future research directions.

## 2. Problem Formalization

This work assumes the availability of a corpus $T^{s}$ encompassing single-channel speech recordings belonging to two classes, i.e., class dictionary D = {*Healthy*, *Depressed*}. Moreover, as per the audio pattern recognition literature [16,17,18], we assume that such mental state associated speech patterns follow consistent, yet unknown probability density functions denoted as $P_{i},i\in D$. We further assume that at each time instance, there is one dominant mental state. Importantly, this work does not assume *a priori* availability of subject-specific knowledge. The final goal is to predict the mental state expressed in novel speech recordings while considering a subject-independent experimental protocol.

## 3. The Proposed Solution

This section presents the pipeline of the proposed methodology, describing the two fundamental processing stages, i.e., feature extraction and audio pattern recognition.

### 3.1. Feature Extraction

Towards capturing diverse aspects of the available speech signals, we employed three feature sets, as described in the following paragraphs.

#### 3.1.1. Mel-Frequency Cepstral Coefficients

The initial set of features is sourced from the speech/speaker recognition paradigm [19], and it has demonstrated effectiveness in various generalized sound classification tasks, encompassing the medical acoustics field [20,21,22]. MFCCs serve as a condensed representation of the spectrogram. To derive these coefficients, the spectrogram undergoes a process where it is initially filtered through the Mel-filterbank to align the frequency scale more closely with the human hearing system. Subsequently, the log operator is applied to appropriately distribute the data, and the information is ultimately condensed through the Discrete Cosine Transform. The first 13 coefficients are kept along with their velocity and acceleration coefficients since their evolution over time could be significant when processing speech signals [23].

#### 3.1.2. Teager Energy Operator Autocorrelation Envelope

The second feature set is derived by processing the speech signals by means of the Teager energy operator [14]. It is tailored to handle speech generated in stressful conditions by effectively modeling the related airflow patterns. Consequently, it incorporates information that may not be encompassed by MFCCs and could prove valuable in analyzing speech indicative of a depressive state, where there may be a significant impact from psychological stress [24].

To obtain the autocorrelation envelope using TEO, the audio signal undergoes an initial filtering process using sixteen critical bands (see Table 1). Following that, the area covered by the autocorrelation is computed for each frame and then divided by half of the frame length for normalization purposes. The resulting dimensionality is sixteen, corresponding to the number of critical bands.

#### 3.1.3. Fundamental Frequency/Pitch and Harmonic Ratio

These features capture periodicity characteristics exhibited in the available speech signals. Their calculation is carried out using the normalized autocorrelation function [25], and they have been effective in various speech processing applications, including stress analysis [26,27], especially in characterizing voiced/unvoiced frames, which may provide useful insights in the present application scenario.

Overall, the structure of the log-mel spectrogram presents presents notable variations over time, while the respective healthy structure exhibits constant behavior. Moreover, the healthy speech includes considerable energy in the higher frequency bands which is well reflected in the values of the area under the Teager Energy Operator autocorrelation envelope.

### 3.2. Universal Probabilistic Model

This section describes the audio pattern recognition stage, which is based on the universal model denoted as M, trained on a balanced subset of the training set. Once M is available, the adaption process follows, where adapted versions of M are constructed, each one representing a specific class. As such, potential class imbalances have only a minor impact on the final model since M’s parameters are estimated on a plethora of data. On top of that, the class-specific models include information representing both the target class and the rest, which otherwise would have remained unseen [28].

**Figure 1:**
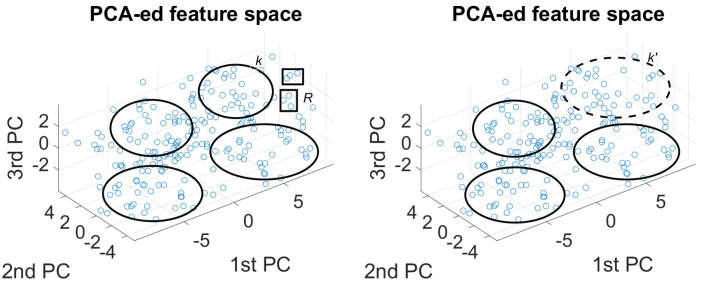
MAP-based adaptation of the *k*-the component of model M using class-specific observations *R*.

In this work, M is fed with balanced subpopulations of the training data so that there is no bias favoring one or more classes, thus avoiding overfitting. Thus, M estimates the distribution of the entire feature space, including both speech classes. Class-specific versions of M are produced via Maximum-a-posteriori adaptation, as shown in Figure 1. As such, the well-learned parameters are suitably adapted to the characteristics of each speech class. In the following, we describe the HMM, the Maximum-a-posteriori adaptation, the sample selection scheme, and the speech depression classification algorithm.

### 3.3. Hidden Markov Model

Universal and class-specific models are HMMs, which are composed of the following elements:

- the number of states *S*,
- the PDF’s approximating states’ distribution via a Gaussian mixture model (GMM), $P\left(x|\theta \right)=\sum_{k=1}^{K}p_{k}p\left(x|\theta_{\left(k\right)}\right)$, where $p_{k}^{\prime }s$ are the weights associated with each mixture, *x* is the feature vector, $\theta_{\left(k\right)}$ denotes the k−th Gaussian mixture, θ=[σ,μ], $p\left(x|\theta_{\left(k\right)}\right)=\frac{1}{{\left(2\pi \right)}^{d/2}\left|\sigma_{k}\right|}e^{-\frac{1}{2}{\left(x-\mu_{k}\right)}^{t}\sigma_{k}^{-1}\left(x-\mu_{k}\right)}$
- the state transition probability matrix $A=\{a_{ij}\}$, where entry $a_{ij}$ comprises the probability of moving from state *j* at time *t* to state *i* at time *t + 1*, and
- the initial state distribution $\pi =\{\bar{\pi_{i}}\}$, where $\bar{\pi_{i}}$ is the probability that HMM starts in state *i*, i.e., $\pi_{i}=P\left[S_{1}\right],1\leq i\leq S$.

Training is performed via the Baum-Welch algorithm based on an Expectation Maximization logic  [29]. During testing, the Viterbi algorithm [30] identifies the most probable series of HMM states.

### 3.4. Correlation-Based *k*-Medoids for Selecting Training and Adaptation Data

In such universal modeling schemes, the selection of training and adaptation data comprises a relevant aspect that may affect modeling efficiency [31]. Thus, this work employs a data organization scheme considering both inter- and intra-class similarities/dissimilarities as assessed by a multidimensional correlation. As such, we used the *k-mediods clustering algorithm*, which unlike the standard *k*-means, is able to incorporate generic pairwise similarity metrics, decreasing the influence of outliers [32]. Motivated by the findings in the sound recognition field [33,34,35], we employed Canonical Correlation Analysis (CCA) to quantify the distance between feature vectors representing mental health status as captured from speech signals. Unlike GMM-based representations [36], correlation-based metrics may be more meaningful from a perceptual point of view.

More in detail, the distance measure between feature vectors $f^{i}$ and $f^{j}$, extracted from speech samples *i* and *j*, respectively, is the canonical correlation. CCA provides two projection planes, i.e., $w_{i}\in R^{d}$ and $w_{j}\in R^{d}$, where *d* is the dimensionality of the feature vectors and where the canonical correlation ρ is maximized:

(1) $$\rho_{i,j}=\frac{w_{i}^{T}f^{i}f^{j,T}w_{j}}{\sqrt{\left(w_{i}^{T}f^{i}f^{i,T}w_{i}\right)\left(w_{j}^{T}f^{j}f^{j,T}w_{j}\right)}}.$$

It is important to note that $\rho_{i,j}$ is independent of the scaling of $w_{i}$ nor $w_{j}$; thus, computing ρ can be formalized as follows:

(2) $$w_{i},w_{j}w_{i}^{T}f^{i}f^{j,T}w_{j},$$

subject to

(3) $$w_{i}^{T}f^{i}f^{i,T}w_{i}=w_{j}^{T}f^{j}f^{j,T}w_{j}=1.$$

Vectors $w_{i}$ and $w_{j}$ can be computed by solving the next optimization problem [37]

(4) $$w_{i}w_{i}^{T}f^{i}f^{j,T}{\left(f^{j}f^{j,T}\right)}^{-1}w_{i},$$

subject to

(5) $$w_{i}^{T}f^{i}f^{i,T}w_{i}=1.$$

Thus, we get $\rho_{i,j}$, which is inversely analogous to the distance between $f^{i}$ and $f^{j}$ used in the *k-mediods clustering algorithm* implemented using the Partitioning Around Medoids [38]. At the same time, such a distance is symmetric. Finally, the most centric speech samples of each class are used for training the Universal HMM; the remaining ones are used during the adaptation process.

### 3.5. Maximum A Posteriori Adaptation (MAP)

*MAP* was employed to adapt M using data representing specific classes. Interestingly, *MAP (a)* emphasizes model components that explain the adaptation data, and *(b)* de-emphasizes the components that do not [39].

Such a two-fold process is performed by updating the means of every Gaussian component. Thus, the model is not confused when processing data belonging to other classes since de-emphasized components will be triggered outputting low log-likelihoods. *MAP* is implemented following the mathematically tractable adaptation scheme described in [39]. An illustrative example of the way *MAP* updates component *k* of M using data observations *R* is demonstrated in Figure 1.

During MAP adaptation, we adopted the process described in [39], where conjugate informative priors are M’s parameters. Accordingly, a mathematically manageable adaptation scheme is developed, where each mean μ in M is updated using the following formula:

(6) $${\hat{\mu }}_{s}^{k}=\frac{N_{s}^{k}}{N_{s}^{k}+\tau }{\bar{\mu }}_{s}^{k}+\frac{\tau }{N_{s}^{k}+\tau }\mu_{s}^{k},$$

where *s* denotes M’s state, *k* the Gaussian component, τ a weighting factor, and $\bar{\mu }$ the mean of the parameters of the adaptation data. *N* represents the occupation likelihood with respect to the adaptation data and is calculated as follows:

(7) $$N_{s}^{k}=\sum_{r=1}^{R}\sum_{t=1}^{T}L_{s}^{k,r}\left(t\right),$$

where *R* is a given part of $T^{d}$ reserved for adaptation purposes with length *T*. The average $\bar{\mu }$ used in Equation (6) characterizing the adaptation data is calculated as follows:

(8) $$\bar{\mu }=\frac{\sum_{r=1}^{R}\sum_{t=1}^{T}L_{s}^{k,r}\left(t\right)o_{t}^{r}}{\sum_{r=1}^{R}\sum_{t=1}^{T}L_{s}^{k,r}\left(t\right)},$$

where $o_{t}^{r}$ corresponds to the available adaptation for observation coming from sequence *r* at time *t*.

As such, if case $N_{s}^{k}$ results in a high value, mixture *k* will be significantly revised via *MAP*. On the contrary, the respective μ will not change.

### 3.6. Classification of Speech Depression

Initially, *MAP* is applied in order to obtain the class-specific models corresponding to healthy and patient classes. Subsequently, classification is performed as in Algorithm 1. During the classification phase, the inputs are the class-specific models $M^{h}$ and $M^{p}$, and the unknown audio signal $y_{t}$, and the outputs are the probability vector $p_{v}$, maximum probability ζ, predicted class, and confidence score *c*. Let us note that $M^{p}$ is the model that was trained on patients, while $M^{h}$ is the model representing healthy subjects. Initially, the algorithm extracts the feature set $f_{t}^{y}$ (line 1, Algorithm 1). Next, the probability vector of $p_{v}$ is initialized (line 2, Algorithm 1). In the following, a *for* loop is executed where $f_{t}^{y}$ is tested against the class-specific models $M^{h}$ and $M^{p}$ outputting the corresponding log-likelihoods to populate $p_{v}$ (lines 3–4, Algorithm 1). The maximum log-likelihood ζ is identified, and the winning class determined (line 5, Algorithm 1). Finally, the confidence score *c* is returned (line 6, Algorithm 1).
**Algorithm 1:** The speech classification algorithm.
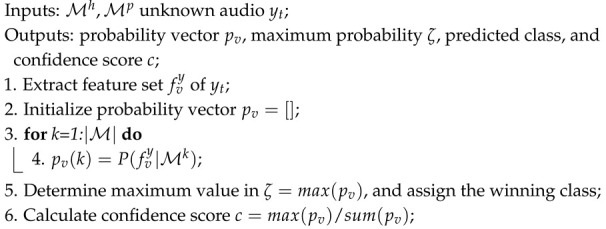


## 4. Experimental Protocol and Analysis of the Results

This section extensively analyzes the (a) employed dataset and figures of merit, (b) parameterization of the proposed approach, (c) experimental results and analysis, (d) model generalization capabilities, and (e) ablation study.

### 4.1. The Employed Dataset

This work employs the Androids dataset [15], which is the only publicly available dataset specifically designed for detecting depression and being thoroughly labeled by medical experts. The corpus includes 228 recordings of 118 native Italian speakers, 64 of whom have been diagnosed with depression. Interestingly, the dataset considers two tasks, i.e., *reading* and *spontaneous* (as captured during an interview) speech. The available recordings last more than 1 h and 30 min for the first task and approximately 7 h and 30 min for the second. The duration associated with female subjects is longer, which reflects the fact that females exhibit depression more frequently than males [40]. The dataset comprises 228 recordings, with 122 originating from depressed participants and 106 from non-depressed participants. The average participants’ age is 47 years, and they all have a similar education level. Such variations are equally considered in both healthy and control subjects so that differences exhibited in their speech patterns can be related to their mental state. Last but not least, the corpus comes with a standardized 5-fold experimental protocol facilitating the reproduction and comparison of competing methodologies. Moreover, the following figures of merit are used: *accuracy*, *precision*, *recall*, and F1 score. More detailed information is available in [15].

### 4.2. Parameterization of the Proposed Solution

The dataset is sampled at 16kHz, and we set the frame and hop size equal to 30 ms and 10 ms, respectively. During the learning of HMMs, the maximum number of iterations with respect to *k*-means initialization, EM, and Baum-Welch algorithms were 50, 25, and 25, respectively [41]. Sets s∈{3,4,5,6,7} and g∈{2,4,8,16,32,64,128} were exhaustively searched in order to find the combination of the number of states and Gaussian components providing the maximum recognition accuracy on a validation set equal to 10% of the training ones.

### 4.3. Experimental Results and Analysis

This subsection reports the depression identification results, adopting the standardized figures of merit in a subject-independent setting. We first present the experimental results obtained with respect to the *Reading Task* and then proceed to the *Interview Task*.

Table 2 includes the results with respect to the *Reading Task*, while the highest rates per figure of merit are emboldened. After the optimization phase, the best-performing UBM-HMM encompassed 3 states, each modeled by 16 Gaussian mixtures, while making use of the proposed data selection scheme. Here, the feature set included the MFCCs along with velocity and acceleration coefficients. The remaining features did not offer improved performance. Such a probabilistic scheme reached an excellent F1 score of 96.2 ± 1.1%. Importantly, the proposed probabilistic scheme significantly outperforms the existing approaches based on Support Vector Machine (SVM) and Long short-term memory (LSTM, one hidden layer with 32 hidden states) models with respect to every figure of merit. The achieved precision (99.1 ± 0.7%) is higher than recall (93.5 ± 0.9%). As such, the system’s depression identifications are reliable, and, at the same time, actual positives were identified correctly. It should be stressed that such a performance is achieved with a relatively simple model of 3 states reflecting the number of stationarity changes exhibited in the available data.

**Figure 2:**
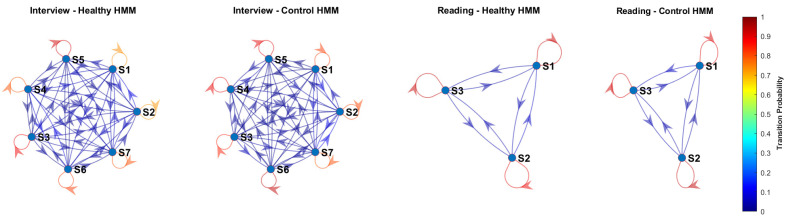
The topologies including the transition probabilities of the HMMs constructed to address Interview and Reading tasks.

The topologies, including the transition probabilities of the constructed HMMs, are illustrated in Figure 2. We see that fully connected HMMs were employed, favoring same-state transitions. It is evident that same-state transition probabilities are higher in speech associated with depression, showing that the respective patterns remain constant for a longer duration compared to healthy speech, as generally expressed by a control subject. In addition, the evolution of the best-performing features does not follow the typical left-right topology in speech recognition, reflecting the different nature of the present problem. We argue that HMM-based modeling of Mel-scaled spectrum summarization, along with its variation over time, comprises a particularly effective way of identifying depression in speech captured during the reading task.

The obtained results with respect to the *Interview Task* are tabulated in Table 3. The optimization based on the validation set yields an ergodic UBM-HMM fed with MFCCs and their velocities, TEO autocorrelation area, fundamental frequency, and harmonic ratio. In this case, the models consist of 7 states and 4 Gaussian components. We observe that the proposed method outperforms the state of the art based on LSTM with respect to all considered figures of merit, reaching an F1-score of 88.9 ± 1.7%. In addition, the achieved recall (92.3 ± 1.8) is higher than precision (85.8 ± 1.9), meaning that the proposed scheme performs well in identifying true positives, while depression identification is not as reliable as during the *Reading Task*. Such a performance behavior is expected to a certain extent since speech recorded during the interviews is spontaneous and exhibits highly non-stationary characteristics when compared to speech recorded during reading. In fact, such a modeling scheme offers important insights into understanding depression classification; additional features and a greater number of states were required to boost the performance, which, nonetheless, did not reach the previous level. The higher number of states (7 vs. 3) implies significantly stronger non-stationary phenomena, clearly demonstrating that the *Interview Task* presents increased difficulty during the modeling phase.

Furthermore, in Figure 2, we observe the fully connected HMM topology and the associated transition probabilities. We see that same-state transitions are still favored; however, the respective probabilities are lower compared to the reading task, which is expected in spontaneous speech, where the exhibited patterns may change at a higher pace. Overall, we argue that the results offered by combining features that capture complementary information along with the universal background modeling scheme are more than satisfactory and provide valuable insights for the problem at hand.

The code to reproduce the experiments is available at https://sites.google.com/site/stavrosntalampiras/, accessed on 16 February 2025.

### 4.4. Testing Model Generalization Capabilities Across Tasks

During the present experimental phase, we examined the generalization capabilities of the best-performing model in a given task over the remaining one. The respective results are presented in Table 4. There, we observe that the model trained on data coming from the *Reading Task* does not generalize as well as the model trained on *Interview Task* data. The rates associated with the *Interview Task* are lower than the respective ones when the same feature set is fed to a larger model. Furthermore, the results obtained in the *Reading Task* are generally higher but present a strong unbalance in precision and recall rates. At the same time, it does not reach the performance level provided by the best-performing model in the *Reading Task*, which may indicate that the additional features capture redundant information while the model includes an unnecessarily large number of states to explain the feature distribution and evolution for the specific task. Overall, the achieved results show that the constructed models are able to generalize up to a certain extent, even though there are relevant differences in the speech data distributions associated with these tasks; as such, transfer learning technologies may be beneficial [42,43].

### 4.5. Ablation Study

This section alters certain components of the proposed approach in order to not only understand their effect on performance but also provide meaningful insights regarding the problem at hand. During the first stage, we examine the influence of the considered features and modeling with respect to the *Reading Task*. Table 5 includes the results achieved with a reduced and augmented feature set, i.e., starting from the sole MFCCs up to MFCCs with their velocity and acceleration, along with the TEO, F0, and HR features. We observe a considerable increase in the reported values since complementary information is captured at each each stage. However, the addition of TEO, F0, and HR to the best-performing feature set (see Table 2) reduced the overall scores, which implies that they do not offer further distinctive information with respect to the present problem. Indeed, stress-capturing features are more relevant in the second task, where the presence of such patterns is evident. It is worth noting that the MFCCs alone achieve a rather robust performance (91.3 ± 2.1%), which may be attributed to the powerful modeling scheme as well. In fact, class-specific HMMs, i.e., without the universal modeling, are able to reach rates similar to those reported in the related literature, confirming the suitability of such a generative classification scheme. We further assessed the effect of topology and data selection scheme. We see that HMMs restricted to left-right transitions provide considerably poorer performance, which shows that such a connection logic does not reflect well the characteristics of the present problem. Moreover, we examined the effectiveness of the proposed data selection scheme and contrasted it with a clustering-based one [36]. Interestingly, such a data selection scheme offered improved balance across precision and recall rates. However, it did not surpass the proposed scheme, which takes into account correlation-based distances.

The second stage of the ablation study is focused on the *Interview Task*. We examined both the composition of the feature set and the parameterization of the HMMs. The respective results are shown in Table 6. There, we see that the mere use of MFCCs does not address the problem in an effective way, reaching an F1-score of 66.7 ± 5.1%. Considering the velocity coefficient significantly improves the performance (78.3 ± 3.8%), while the TEO information boosts it further, confirming the hypothesis that stress-related characteristics play an important role during the specific task. At the same time, it becomes clear that the selected features carry complementary information strongly related to the problem at hand. In addition, we assessed the effect of the universal modeling scheme, topology, as well as the train/adaptation data selection process. We see the class-specific HMMs reach a smaller F1-score (75.8 ± 4.3%); the left-right topology is unsuitable for modeling depression in speech, while the clustering-based data selection scheme offers satisfactory performance. Overall, the proposed fully-connected UBM-HMM with correlation-based data selection provides robust performance. Importantly, such a model encodes knowledge characterizing both classes and is able to emphasize and de-emphasize components associated with a specific class, thus reducing the amount of misclassifications with respect to the typical class-specific HMMs.

**Figure 3:**
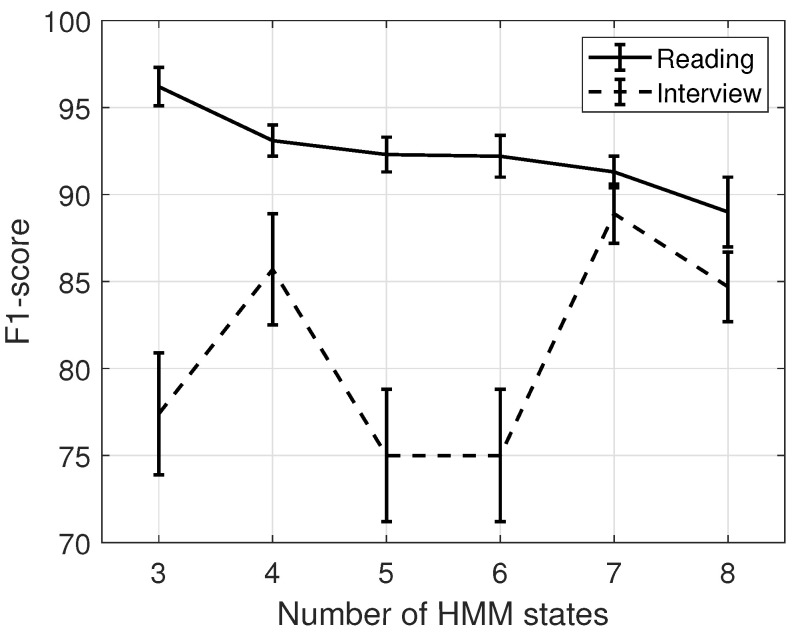
Effect of the number of HMM states on F1-score for Reading and Interview tasks.

Finally, we assessed how the number of states influences performance as expressed in terms of the F1-score. Figure 3 illustrates these alterations for both reading and interview tasks. Overall, the number of states per task is the optimal number of stationarity changes from a healthy/control discrimination point of view. In the former task, we observe a relative decrease in performance as the number of states increases beyond 3, showing that the present non-stationarities are captured better with a small number of states. On the contrary, more states are beneficial for the latter task, where performance is maximized when 7 states are considered. This confirms the hypothesis that processing spontaneous speech is generally a harder task than reading speech, as it presents stronger non-stationary characteristics.

## 5. Interpretation of the Model’s Predictions and Interaction with Medical Experts

This section highlights the importance of having available clear interpretation of the entire AI pipeline, especially when it comes to critical medical settings. In addition, we describe the ways that the proposed system is able to interact with the medical experts and build trust towards bridging the usability gap existing between AI-based tools and domain experts.

Importantly, this work applies the explainability-by-design concept in the medical acoustics field. In other words, each system component is designed following the problem specifications and characteristics of the *a priori* available knowledge. As such, the feature set composition was determined along with the model choice and parameterization. The present framework exploits handcrafted features that are easily traced back to the signal’s structure, combined with a generative classifier able to provide interpretable predictions. Interestingly, HMMs can point out depression-related information in a speech segment as well as its evolution over time. Unlike NNs, which behave as black boxes, their operation is fully transparent and governed by clear probabilistic rules [44,45]. They are (a) verifiable, i.e., it can be proved that they process inputs correctly, (b) reliable, i.e., there is an expected operation for inputs located in and out of the known distribution (c) auditable, i.e., we are able to examine their internal state and explain their predictions, and (d) free of bias, since they are trained on correlation-based balanced data subpopulations, thus do not exhibit any type of preferences.

**Figure 4:**
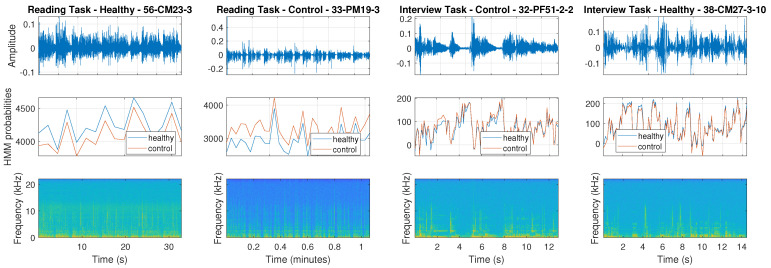
The probabilities output by the UBM-HMM on recordings representing both Healthy and Control subjects with respect to Reading and Interview tasks.

Figure 4 illustrates the audio waveforms and output probabilities of the UBM HMM when processing recordings and spectrograms representing both healthy and control subjects with respect to reading and interview tasks. Interestingly, the proposed scheme follows a short-time based processing approach and provides a time series of probabilities characterizing each speech segment. Therefore, the medical expert could monitor the evolution of the subject’s status over time and gain valuable insights regarding her/his condition. In the first and third figures, we observe the way the model processes speech coming from healthy subjects, while the second and fourth figures come from control ones. The first pair was recorded during the execution of the reading task, while the second during the interview one. The produced probabilities correspond well with the ground truth, and segments with high confidences are highlighted to the expert.

Overall, we observe that the probability differences of the two models are higher for the first task, where the model provided higher F1-scores (see Table 2 and Table 3). Importantly, the UBM HMM operates in a transparent way, where one is able to monitor the effective state path along with the emitted probabilities and the resulting prediction based on the maximum a posteriori probability. Moreover, at any given point in time, we can compare the probabilities and assign the winning class. As such, even though the data are only annotated at the recording level, they can be meaningfully segmented into healthy/depressed parts of interest, while the models provide an overall picture of the speech content from the beginning to the end of the session. At the same time, the overall approach is characterized by relatively low computational complexity both during training and testing time.

Interestingly, the proposed framework not only provides an interpretable probability-based representation of a specific speech segment but may also interact with medical experts to improve familiarization and build trust, thus filling an existing literature gap [46,47]. Once the experts become familiar with such an AI-based tool and understand in-depth its operation, they may utilize it effectively and improve and customize its output. The framework may answer to questions, such as (a), which segments contributed most to the prediction, (b) find similar segments from the same same or other patients, etc.

To respond, the proposed framework isolates segments that maximze the confidence metric so that the medical expert may listen only to those parts, specifically during the diagnoses process. Subsequently, it may provide similarity with segments coming from different subjects with confirmed diagnosis. Similar segments can be easily identified by comparing the effective HMM path and the range of the emitted probabilities. As such, the expert may decide to follow a similar/personalized treatment. In addition, the framework may calculate and store both short- and long-term statistics for browsing at a later stage to assist the diagnosis process. Such a Q&A-based interaction scheme may effectively assist the use and acceptance of AI-based tools by the medical personnel. Overall, we argue that the proposed approach not only comprises a highly potent modeling scheme but, at the same time, offers clear interpretations that are strongly required in such a critical applications.

## 6. Conclusions

This article presents an automatic approach for identifying depression in speech. Two tasks were considered, i.e., *Reading* and *Interview*, for which different features are employed based on the characteristics of the recorded signals. A diverse feature set was formed, capturing complementary aspects of the signals’ structures. These are fed to a universal modeling scheme able to encode existing temporal dependencies while suitably selecting training and adaptation datasets via a suitably designed correlation-based algorithm. Interestingly, such a scheme incorporates knowledge coming from both healthy and control subjects and outperforms the state of the art with respect to both tasks. At the same time, the present approach can be extended in a straightforward way and operate under non-stationary environments, i.e., it may encompass additional mental states as long as the corresponding data become available without requiring complete retraining. Last but not least, an appropriate Q&A-based interaction scheme (see Section 5) was described, able to meaningfully assist medical professionals during the diagnosis process. The incorporation of such a feature could enhance the adoption of AI-driven solutions within the medical field, as it enables experts to comprehend the rationale behind each prediction. The integration of such features not only aids in closing the usability loop but also supports the education of young psychiatrists and medical personnel.

The present design choices were motivated by the fact that clear, interpretable decisions are required in the medical field; otherwise, there is a risk that such tools will not be adopted by domain experts. In fact, the proposed system is fully interpretable, i.e., the operation of every component is clear and transparent, unlike black-box systems where an architecture may provide high accuracy without clear reasoning. Actually, such works attempt to explain their operation by employing a reverse logic, where they are trying to understand why an architecture performs well at a post-evaluation phase [47] by analyzing the relevance/weights that the architecture assigns to specific input regions, and thus interpret them in an opportunistic way, which might not reflect well the characteristics of the problem at hand. As an additional consideration, in many cases such architectures may focus on regions that do not contain expected constituent frequencies but help distinguish between classes, which complicates or renders any interpretation efforts ineffective. That said, there are methodologies targeting the interpretability of the trained weights of the different components in a deep architecture [48]. However, HMMs usually have much fewer parameters, which significantly eases the interpretability process. We argue that medical AI frameworks [46], unless accompanied by a rigid etiology framework, have very little chance of being widely adopted in such critical medical applications, especially when considering the recent requirements imposed by the AI Act (REGULATION (EU) 2024/1689 OF THE EUROPEAN PARLIAMENT AND OF THE COUNCIL).

In the subsequent phases of this research, our intention is to explore the following facets:

- consider hybrid HMMs, i.e., with emission probabilities based on neural networks [49],
- modify the current framework to suit applications with similar specifications,
- integrate additional mental states and develop appropriate sets of features,
- explore temporal integration methodologies (statistics, spectral moments, autoregressive models, etc.) at the feature level, given that mental states tend to not change rapidly over time,
- examine the efficiency of identification in small data environments, specifically addressing scenarios with limited data availability, such as having few or even just one training sample per class, and
- improve the capabilities of the interpretability module, with a particular emphasis on ensuring user-friendliness and garnering acceptance among medical experts.

## Figures and Tables

**Table 1:** The frequency bands used for the extraction of the Teager Energy Operator autocorrelation envelope.

Band ID	Lower (Hz)	Center (Hz)	Upper (Hz)
1	100	250	400
2	400	500	600
3	600	700	800
4	800	910	1020
5	1020	1140	1260
6	1260	1400	1540
7	1540	1690	1840
8	1840	2000	2160
9	2160	2350	2540
10	2540	2750	2960
11	2960	3200	3440
12	3440	3720	4000
13	4000	4310	4620
14	4620	5010	5400
15	5400	5850	6300
16	6300	6850	7400

**Table 2:** The results of the proposed method versus the state of the art on detecting depression on the Reading Task. The highest rates per figure of merit are emboldened.

Reading Task				
Approach	Acc.	Prec.	Rec.	F1
Random	50.1	51.8	51.8	51.8
UBM-HMM	**96.2 ± 1.2**	**99.1 ± 0.7**	**93.5 ± 0.9**	**96.2 ± 1.1**
Linear SVM	69.6 ± 5.3	73.6 ± 19.1	68.8 ± 12.0	68.4 ± 7.7
LSTM [15]	84.4 ± 1.1	84.5 ± 2.1	85.6 ± 2.8	83.7 ± 1.1

**Table 3:** The results of the proposed method versus the state of the art on detecting depression on the Interview Task. The highest rates per figure of merit are emboldened.

Interview Task				
Approach	Acc.	Prec.	Rec.	F1
Random	50.5	55.2	55.2	55.2
UBM-HMM	**87.0 ± 1.2**	**85.8 ± 1.9**	**92.3 ± 1.8**	**88.9 ± 1.7**
Linear SVM	73.3 ± 10.6	73.5 ± 16.1	74.5 ± 13.2	73.6 ± 13.6
LSTM [15]	83.9 ± 1.3	85.8 ± 3.1	86.1 ± 2.7	84.7 ± 0.9

**Table 4:** Model generalization across tasks.

Acc.	Prec.	Rec.	F1
**Interview Task**			
68.5 ± 5.9	73.3 ± 5.4	66.9 ± 4.8	70.0 ± 6.1
**Reading Task**			
80.0 ± 3.7	76.5 ± 4.1	99.2 ± 0.5	86.4 ± 3.5

**Table 5:** The results associated with the conducted ablation study with respect to the Reading Task.

Reading Task				
Approach	Acc.	Prec.	Rec.	F1
UBM-HMM MFCC	91.3 ± 1.9	99.0 ± 0.8	84.6 ± 3.4	91.2 ± 2.1
UBM-HMM MFCC_D	92.1 ± 2.1	99.1 ± 0.9	85.7 ± 3.1	91.9 ± 2.5
UBM-HMM MFCC_D_A	**96.2 ± 1.2**	**99.1 ± 0.7**	**93.5 ± 0.9**	**96.2 ± 1.1**
UBM-HMM MFCC_D_A_TEO	92.4 ± 2.2	99.1 ± 0.9	87.0 ± 2.5	92.6 ± 1.8
UBM-HMM MFCC_D_A_TEO_F0_HR	93.1 ± 2.1	99.1 ± 0.9	88.2 ± 1.9	93.3 ± 1.7
class-specific HMM MFCC_D_A	85.3 ± 3.2	84.2 ± 2.9	86.1 ± 3.6	85.1 ± 2.9
left-right UBM-HMM MFCC_D_A	83.8 ± 4.1	83.9 ± 3.5	82.8 ± 4.3	83.3 ± 3.9
clustering UBM-HMM MFCC_D_A	95.0 ± 1.8	95.8 ± 2	93.9 ± 2.3	94.8 ± 1.5

**Table 6:** The results associated to the conducted ablation study with respect to the Interview Task.

Interview Task				
Approach	Acc.	Prec.	Rec.	F1
UBM-HMM MFCC	69.6 ± 4.7	87.5 ± 5.3	53.8 ± 4.5	66.7 ± 5.1
UBM-HMM MFCC_D	78.3 ± 4.2	90.0 ± 4.4	69.2 ± 3.9	78.3 ± 3.8
UBM-HMM MFCC_D_A	79.6 ± 4	92.4 ± 4.2	70.3 ± 3.8	79.8 ± 3.5
UBM-HMM MFCC_D_A_TEO	84.6 ± 3.5	**95.4 ± 2.7**	71.3 ± 2.6	81.6 ± 3.1
UBM-HMM MFCC_D_TEO_F0_HR	**87.0 ± 1.2**	85.8 ± 1.9	**92.3 ± 1.8**	**88.9 ± 1.7**
class-specific HMM MFCC_D_A_TEO_F0_HR	77.4 ± 5.1	84.3 ± 4.7	68.9 ± 5.2	75.8 ± 4.3
left-right UBM-HMM MFCC_D_A_TEO_F0_HR	84.3 ± 2.7	85.1 ± 2.8	80.9 ± 2.4	82.9 ± 2.2
clustering UBM-HMM MFCC_D_A_TEO_F0_HR	88.5 ± 2.4	88.1 ± 1.9	88.6 ± 2	87.3 ± 2.1

## Data Availability

The dataset is available in [15].
